# Influence of Lipid A Acylation Pattern on Membrane Permeability and Innate Immune Stimulation

**DOI:** 10.3390/md11093197

**Published:** 2013-08-26

**Authors:** Yanyan Li, Zhou Wang, Jiuzhou Chen, Robert K. Ernst, Xiaoyuan Wang

**Affiliations:** 1 State Key Laboratory of Food Science and Technology, Jiangnan University, Wuxi 214122, China; E-Mails: yanyanli1123@hotmail.com (Y.L.); wangzhou0920@126.com (Z.W.); jiuzhou1103@163.com (J.C.); 2 Department of Microbial Pathogenesis, University of Maryland Dental School, Baltimore, MD 21201, USA; E-Mail: rkernst@umaryland.edu

**Keywords:** endotoxin, lipid A, lipopolysaccharide, TLR4/MD2, membrane permeability, PagL

## Abstract

Lipid A, the hydrophobic anchor of lipopolysaccharide (LPS), is an essential component in the outer membrane of Gram-negative bacteria. It can stimulate the innate immune system via Toll-like receptor 4/myeloid differentiation factor 2 (TLR4/MD2), leading to the release of inflammatory cytokines. In this study, six *Escherichia coli* strains which can produce lipid A with different acylation patterns were constructed; the influence of lipid A acylation pattern on the membrane permeability and innate immune stimulation has been systematically investigated. The lipid A species were isolated and identified by matrix assisted laser ionization desorption-time of flight/tandem mass spectrometry. *N*-Phenyl naphthylamine uptake assay and antibiotic susceptibility test showed that membrane permeability of these strains were different. The lower the number of acyl chains in lipid A, the stronger the membrane permeability. LPS purified from these strains were used to stimulate human or mouse macrophage cells, and different levels of cytokines were induced. Compared with wild type hexa-acylated LPS, penta-acylated, tetra-acylated and tri-acylated LPS induced lower levels of cytokines. These results suggest that the lipid A acylation pattern influences both the bacterial membrane permeability and innate immune stimulation. The results would be useful for redesigning the bacterial membrane structure and for developing lipid A vaccine adjuvant.

## 1. Introduction

Gram-negative bacteria contain inner and outer membranes. The inner membrane is composed of phospholipids, while the outer membrane is an asymmetric lipid bilayer with an inner leaflet of phospholipids and an outer leaflet of lipopolysaccharide (LPS). This asymmetric lipid bilayer serves as a permeation barrier to maintain optimal membrane fluidity and facilitate proper cellular function [1,2]. LPS is structurally divided into three parts: *O*-antigen, core saccharide, and lipid A [3]. Lipid A, the hydrophobic anchor of LPS, is the biologically active component and can induce proinflammatory responses through Toll-like receptor 4/myeloid differentiation factor 2 (TLR4/MD2) on the surface of immune cells [4]. Lipid A with different structures could stimulate different signal transduction pathways via TLR4/MD2 and two sets of adaptor proteins, myeloid differentiation primary response gene 88 (MyD88) and the Toll/Il-1 receptor (TIR)-domain-containing adaptor-inducing interferon-β (TRIF). The pathway through MyD88 can produce high levels of inflammatory cytokines such as tumor necrosis factor alpha (TNF-α) and interleukin-8 (IL-8), while the TRIF mediated pathway can produce low levels of inflammatory cytokines [5,6]. Lipid A with only one phosphate group selectively activates the TRIF mediated pathway [7]. The differential stimulation ability of lipid A via TLR4/MD2 is associated with several bacterial diseases [8]. 

*E. coli* lipid A is a disaccharide of glucosamine phosphorylated at 1 and 4′-positions and acylated at the 2-, 3-, 2′-, and 3′-positions with *R*-3-hydroxymyristate. The OH groups of the *R*-3-hydroxymyristate chains at 2′- and 3′-positions are further acylated with laurate and myristate, respectively. This relatively conserved hexaacylated lipid A can be recognized by the TLR4/MD2 complex and induce a strong proinflammatory response, leading to secretion of cytokines [9]. In some Gram-negative bacteria, additional covalent modifications of lipid A occurs, leading to changes in the length and number of fatty acids, the number of phosphate and carbohydrate moieties [10,11]. The structural variation of lipid A can alter the stimulation ability of LPS via TLR4/MD2. In *Yersinia pestis*, the lipid A acylation pattern is regulated by temperature. When grown at 37 °C, *Y. pestis* lipid A is tetraacylated and showed stronger stimulation to murine immune cells than to human immune cells; when grown at lower temperatures, *Y. pestis* lipid A is mainly hexaacylated and showed equal stimulation ability to human and mouse immune cells [12,13]. In *Salmonella enterica*, the structure of lipid A is modified in response to environmental conditions by several enzymes under the control of the two-component regulatory system PhoP-PhoQ [14,15]. This lipid A modification is related to *Salmonella* virulence [16]. PhoQ, a sensor histidine kinase, responds to specific environmental signals through phosphorylating PhoP, leading to the activation and repression of different genes, including the lipid A modification gene *pagL* which encodes a lipid A 3-*O*-deacylase located in the outer membrane [17], and may modulate the lipid A signaling through TLR4/MD2 [18].

In most Gram-negative bacteria, LPS has been found to play an important role in membrane permeability, cell adhesion and stability. For further understanding the influence of the acylation pattern of lipid A on innate immune recognition and stimulation, six *E. coli* strains that can synthesize lipid A with different acylation patterns were constructed. The structures of lipid A in these strains were confirmed by matrix assisted laser ionization desorption-time of flight/tandem mass spectrometry MALDI-TOF/MS. The effects of the lipid A made by theses strains on the membrane permeability and the innate immune stimulation were characterized. 

## 2. Results and Discussion

### 2.1. Structure Analysis of Lipid A Extracted from Six *E. coli* Strains

**Figure 1:**
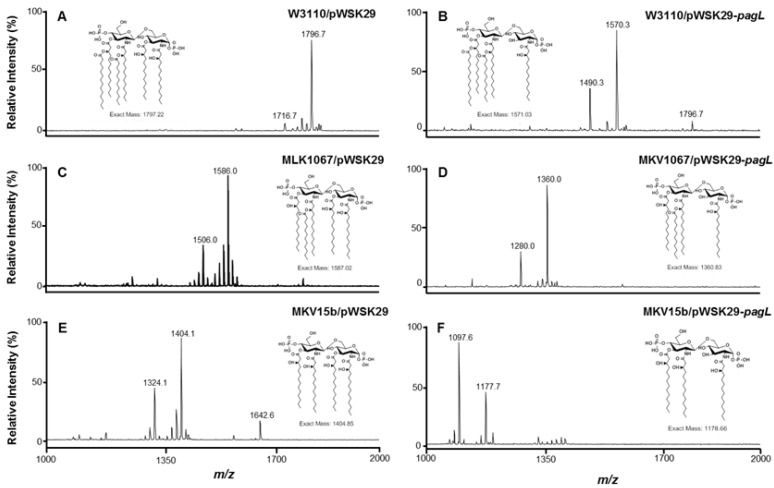
Negative ion MALDI-TOF MS of lipid A purified from *E. coli* strains W3110/pWSK29 (**A**), W3110/pWSK29-*pagL* (**B**), MLK1067/pWSK29 (**C**), MLK1067/pWSK29-*pagL* (**D**), MKV15b/pWSK29 (**E**), and MKV15b/pWSK29-*pagL* (**F**).

Three strains were constructed by overexpressing the *S. typhimurium* *pagL* gene in *E. coli* W3110, MLK1067 and MKV15b, respectively. The *pagL* gene encodes deacylase PagL which can selectively remove the acyl chain from the 3-position of lipid A. W3110 can synthesize the hexaacylated lipid A; MLK1067, a *lpxM* mutant, can synthesize pentaacylated lipid A [19]; MKV15b, a *lpxL*, *lpxM*, *lpxP* triple mutant, can synthesize tetraacylated lipid A [20]. MKV15b is a spontaneous revertant of the parental triple-knockout strain MKV15, but unlike the parental strain, MKV15b grows well in Luria-Bertani broth at 37 °C [21]. To test if PagL functions in these different *E. coli* strains, lipid A was isolated from W3110/pWSK29, W3110/pWSK29-*pagL*, MLK1067/pWSK29, MLK1067/pWSK29-*pagL*, MKV15b/pWSK29, MKV15b/pWSK29-*pagL*, respectively, and analyzed using MALDI-TOF MS (Figure 1).

A major peak at *m*/*z* 1796.8 was observed in the spectrum of lipid A isolated from the control strain W3110/pWSK29, corresponding to the molecular ion [M − H]^−^ of the hexaacylated lipid A [20]. The minor peak at *m*/*z* 1716.7 is derived from the molecular ion by loss of a phosphate during the process (Figure 1A). Two major peaks at *m*/*z* 1570.6 and 1490.3 were observed in the spectrum of lipid A isolated from W3110/pWSK29-*pagL* (Figure 1B). The peak at *m*/*z* 1570.6 resulted from the deacylation of hexaacylated lipid A by PagL, and the peak at *m*/*z* 1490.3 is derived by further loss of a phosphate. The minor peak at *m*/*z* 1796.7 indicates that there is small amount of hexaacylated lipid A not deacylated by PagL in *E. coli* W3110/pWSK29-*pagL*.

Two major peaks at *m*/*z* 1586.0 and 1506.0 were observed in the spectrum of lipid A isolated from MLK1067/pWSK29 (Figure 1C). The peak at *m*/*z* 1586.0 corresponds to the molecular ion [M − H]^−^ of the pentaacylated lipid A; the peak at *m*/*z* 1506.0 is derived from the molecular ion by loss of a phosphate. Two major peaks at *m*/*z* 1360.0 and 1280.0 were observed in the spectrum of lipid A isolated from MLK1067/pWSK29-*pagL* (Figure 1D). The *m*/*z* values of these two peaks are less than the two peaks at *m*/*z* 1586.0 and 1506.0 of lipid A from MLK1067/pWSK29 by 226 amu, respectively, suggesting they were tetraacylated lipid A species, resulting from the PagL deacylation. 

Two major peaks at *m*/*z* 1404.1 and 1324.1 were observed in the spectrum of lipid A isolated from MKV15b/pWSK29 (Figure 1E). The peak at *m*/*z* 1404.0 corresponds to the molecular ion [M − H]^−^ of the tetraacylated lipid A; the peak at *m*/*z* 1324.1 is derived from the molecular ion by loss of a phosphate [20]. Additionally, the peak at *m*/*z* 1642.6 was observed and corresponds to the modification of a palmitate (C16) at the 3 position by PagP. Two major peaks at *m*/*z* 1177.7 and 1097.6 were observed in the spectrum of lipid A isolated from MKV15b/pWSK29-*pagL* (Figure 1F). The *m*/*z* values of these two peaks are less than the two peaks at *m*/*z* 1404.1 and 1324.1 of lipid A from MKV15b/pWSK29 by 226 amu, respectively, suggesting they were triacylated lipid A species, resulting from the PagL deacylation. 

These data indicate that the *S. typhimurium* *pagL* gene functions well in *E. coli* W3110, MLK1067 and MKV15b, and almost all the lipid A in these strains can be modified by PagL. 

### 2.2. Effects of Lipid A Acylation Pattern on the Membrane Permeability of Different *E. coli* Strains

As major molecules in the outer membranes, the strong lateral interactions between lipid A molecules play an important role in maintaining the outer membrane permeability. Therefore, the change of the acylation patterns of lipid A might influence the permeability of the outer membranes of bacteria. To answer this question, membrane permeability of *E. coli* strains W3110/pWSK29, W3110/pWSK29-*pagL*, MLK1067/pWSK29, MLK1067/pWSK29-*pagL*, MKV15b/pWSK29, and MKV15b/pWSK29-*pagL* was individually determined using *N*-Phenyl naphthylamine (NPN) assay and antibiotic susceptibility test, respectively.

**Figure 2:**
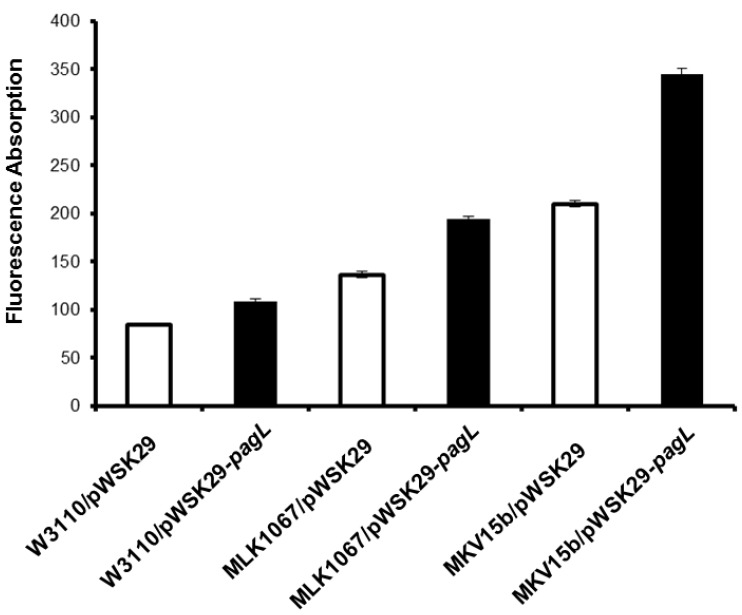
Outer membrane permeability of six *E. coli* strains.

The nonpolar probe NPN tends to stay in hydrophobic environments and fluoresces strongly in hydrophobic environments but weakly in aqueous environments. The membrane permeability of the six *E. coli* strains determined by the NPN assay is shown in Figure 2. After treatment with NPN, the fluorescence absorption for W3110/pWSK29 cells was the lowest, while that for MKV15b/pWSK29-*pagL* cells is 4-fold higher, the highest among the six *E. coli* strains. The fluorescence absorption for cells of W3110/pWSK29, W3110/pWSK29-*pagL*, MLK1067/pWSK29, MLK1067/pWSK29-*pagL*, MKV15b/pWSK29 and MKV15b/pWSK29-*pagL* gradually increases, suggesting that the membrane permeability of *E. coli* cells increases with the decrease of the number of acyl chains of lipid A.

Lipid A molecules tightly packed in the outer membrane form a barrier to antibiotics. Therefore, *E. coli* *lpxA*, *lpxC*, *lpxD*, *lpxL* and *lpxM* mutants are susceptible to hydrophobic antibiotics [22,23]. The susceptibility to different antibiotics of the six *E. coli* strains constructed in this study was determined using disk diffusion assay (Table 1). Similar susceptibility to aztreonam, minocycline, polymyxin B and vancomycin were observed for all the six *E. coli* strains, but their susceptibility to trimethoprim, gentamicin, cefotaxime, impenem and erythromycin were different (Table 1). Overall, W3110/pWSK29 cells were the most resistant, while MKV15b/pWSK29-*pagL* cells were the most sensitive ones among the six *E. coli* strains. The antibiotic susceptibility of *E. coli* cells of six different strains slightly increases with the decrease of the number of acyl chains of lipid A. The results suggest again that the number of acyl chains in lipid A plays an important role in the membrane permeability. 

### 2.3. Effects of Lipid A Acylation Pattern on Innate Immune System Recognition

**Figure 3:**
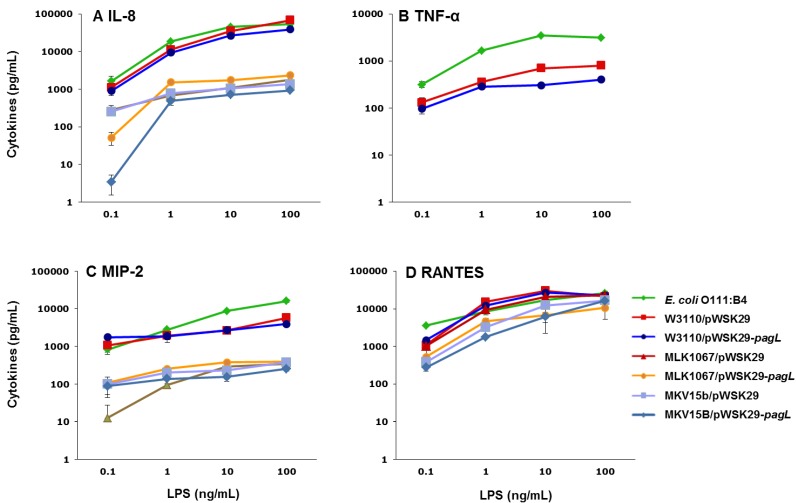
Human interleukin-8 (IL-8) (**A**), tumor necrosis factor alpha (TNF-α) (**B**) and regulated and normal T cell expressed and secreted (RANTES)(**D**) production by THP-1 cells and macrophage inflammatory protein-2 (MIP-2) (**C**) production by MH-S cells after stimulation with lipopolysaccharide (LPS) isolated from different *E. coli* strains.

Lipid A is the biologically active component of LPS. The recognition of lipid A by TLR4/MD-2 complex initiates the proinflammatory responses [24]. It is interesting to know how the acylation pattern of lipid A influences the recognition of TLR4/MD-2 complex. Therefore, LPS were purified from the *E. coli* strains of W3110/pWSK29, W3110/pWSK29-*pagL*, MLK1067/pWSK29, MLK1067/pWSK29-*pagL*, MKV15b/pWSK29 and MKV15b/pWSK29-*pagL*, respectively. LPS with concentrations from 0.1 to 100 ng/mL were incubated with the human monocytic THP-1 cell line and the murine macrophage MH-S cell, respectively, and levels of the MyD88-dependent cytokines IL-8 and TNF-α and the TRIF-dependent cytokines regulated and normal T cell expressed and secreted (RANTES) and macrophage inflammatory protein-2 (MIP-2) were determined by enzyme-linked immunosorbent assay (ELISA) (Figure 3). 

**Table 1:** Antibiotics susceptibility of different *E. coli* strains. Zone of clearing diameter (mm) of disk diffusion test were listed. The diameter of the disk is 6 mm, and the diameter of the zone of clearing shown includes the disk diameter. Each experiment was repeated three times, and the average values are listed here. The error for the three duplicates was usually less than 2 mm.

Antibiotics	W3110/pWSK29	W3110/pWSK29-*pagL*	MLK1067/pWSK29	MLK1067/pWSK29-*pagL*	MKV15b/pWSK29	MKV15b/pWSK29-*pagL*
Trimethoprim	25	24	26	25	28	30
Gentamicin	23	23	24	26	25	28
Cefotaxime	34	34	35	36	36	38
Imipenem	28	29	31	32	32	35
Erythromycin	8	10	16	16	17	18
Aztreonam	34	34	34	34	36	37
Minocycline	16	16	17	18	18	18
Polymyxin B	17	17	18	18	18	20
Vancomycin	6	6	6	6	7	8

*E. coli* O111:B4 LPS, the positive control, induced the highest levels of cytokines in most experiments. Different levels of cytokines were induced by LPS from the six *E. coli* strains, ranging from the high level by LPS from W3110/pWSK29 to the low level by LPS from MKV15b/pWSK29-*pagL*. The levels of IL-8 decrease with the decrease of the number of the acyl chains in lipid A of LPS used for stimulation (Figure 3A). After stimulation by 10 ng/mL LPS, 35,448, 1075 and 1042 pg/mL IL-8 were induced by LPS from W3110/pWSK29, MLK1067/pWSK29 and MKV15b/pWSK29, respectively. Even though they contain the same number of fatty acyl chains, different levels of IL-8 were induced by LPS from W3110/pWSK29-*pagL* and MLK1067/pWSK29, respectively; the former was 26,608 pg/mL and the latter was 1075 pg/mL, suggesting the acylation pattern of LPS is important for the recognition by TLR4/MD2 (Figure 3A). The similar overall pattern was observed for levels of TNF-α after cell stimulation by LPS from the six *E. coli* strains although the exact values are much smaller than that of IL-8 (Figure 3B). Concentrations of TNF-α after stimulation were 699 pg/mL and 307 pg/mL for W3110/pWSK29 and W3110/pWSK29-*pagL*, respectively; whereas the production of TNF-α after stimulation of LPS from MLK1067/pWSK29, MLK1067/pWSK29-*pagL*, MKV15b/pWSK29 and MKV15b/pWSK29-*pagL*, respectively, was almost completely abolished (Figure 3B). The clear difference between the levels of TNF-α after stimulation of LPS from W3110/pWSK29-*pagL* and MLK1067/pWSK29 suggests again that the acylation pattern of LPS is important for the recognition by TLR4/MD2. The overall pattern for levels of RANTES after cell stimulation by LPS from the six *E. coli* strains is similar to that of IL-8 (Figure 3D). The concentrations of RANTES after cell stimulation by LPS fromm W3110/pWSK29 and W3110/pWSK29-*pagL* are higher than LPS from MLK1067/pWSK29, MLK1067/pWSK29-*pagL*, MKV15b/pWSK29 and MKV15b/pWSK29-*pagL* (Figure 3C). MIP-2 production after stimulation of murine macrophage MH-S cell by LPS from the six *E. coli* strains is similar (Figure 3C), but concentrations of MIP-2 after stimulation by LPS from W3110/pWSK29 LPS and W3110/pWSK29-*pagL* are slightly higher (Figure 3C). After stimulation by 10 ng/mL LPS, the production of MIP-2 for W3110/pWSK29, MLK1067/pWSK29 LPS, MKV15b/pWSK29 were 30,046, 20,555 and 12,264 pg/mL, respectively. These results indicate that both the acylation pattern and the number of acyl chains in the lipid A of LPS play important roles for the recognition by TLR4/MD2. 

## 3. Experimental Section

### 3.1. Bacterial Strains, Plasmid and Growth Conditions

Bacterial strains and plasmids used in this study are listed in Table 2. All strains were grown at 37 °C in Luria-Bertani media (10 g/L tryptone, 5 g/L yeast extract, 10 g/L NaCl). When required, ampicillin, kanamycin, and chloramphenicol was added at a final concentration of 100 μg/mL, 30 μg/mL, and 25 μg/mL, respectively.

The expression plasmid pWSK29-*pagL* was constructed. Briefly, the *pagL* was amplified from the genomic DNA of *S. typhimurium*. The forward primer was 5′-GCTCTAGAGGTGGAGTGTATATGAAGAG-3′ in which an XbaI restriction site (underlined) was designed. The reverse primer was 5′-CGGGATCCCTCGCTAATTGTTATTCAAC-3′ in which a BamHI restriction site (underlined) was designed. The polymerase chain reaction product was digested with XbaI and BamHI and ligated into pWSK29. The ligation mixture was transformed into *E. coli* DH5α, and the positive transformants, designated DH5α/pWSK29-*pagL*, and was selected on LB plates containing 100 mg/L ampicillin. For expression in *E. coli*, the pWSK29-*pagL* was then transformed into *E. coli* W3110, *E. coli* MLK1067, *E. coli* MKV15b. 

**Table 2:** Bacterial strains and plasmids used in this work.

Strains or plasmids	Relevant characteristics	Reference or source
Strains		
DH5α	*E. coli* component cells	Invitrogen
*E. coli* W3110	*E. coli F^−^rph-1 IN(rrnD–rrnE)1 λ^−^*	[19]
*E. coli* MLK1067	*E. coli* W3110 *msbB*::*Ωcam*	[19]
*E. coli* MKV15b	*E. coli* W3110 *lpxM*::*Ωcam lpxP::Ωkan lpxL::Tn10*	[20,21]
W3110/pWSK29	W3110 harboring pWSK29	This work
MLK1067/pWSK29	MLK1067 harboring pWSK29	This work
MKV15b/pWSK29	MKV15b harboring pWSK29	This work
W3110/pWSK29-*pagL*	W3110 harboring pWSK29-*pagL*	This work
MLK1067/pWSK29-*pagL*	MLK1067 harboring pWSK29-*pagL*	This work
MKV15b/pWSK29-*pagL*	MKV15b harboring pWSK29-*pagL*	This work
Plasmids		
pWSK29	Low copy vector, Amp^r^	[25]
pWSK29-*pagL*	pWSK29 containing *pagL*	This work

### 3.2. LPS and Lipid A Isolation

*E. coli* LPS were extracted using a hot phenol/water extraction method as described [26]. Further treatment of LPS with RNase A, DNase I, and proteinase K ensured removal of contaminating nucleic acids and proteins [19]. Subsequently, LPS samples were additionally subjected to Folch and Vogel extractions to remove contaminating phospholipids and TLR-2 agonist contaminating proteins [27,28]. 

Lipid A was isolated after hydrolysis in 1% sodium dodecyl sulfate (SDS) at pH 4.5 as described [29]. Briefly, 500 μL of 1% SDS in 10 mM sodium-acetate, pH 4.5, were added to a lyophilized sample. Samples were incubated at 100 °C for one hour and lyophilized. The dried pellets were washed in 100 μL of water and 1 mL of acidified ethanol (100 μL 4 N HCl in 20 mL 95% ethanol). Samples were centrifuged at 5000 rpm for five minutes. The lipid A pellet was further washed three times in 1 mL of 95% ethanol. The entire series of washes was repeated twice. Samples were resuspended in 500 μL of water, frozen on dry ice, and lyophilized. 

### 3.3. Mass Spectrometry Procedures

Negative ion MALDI-TOF/TOF MS experiments was performed as described elsewhere [30]. Briefly, lipids were solubilized in 100 μL chloroform/methanol (2:1, v/v) and spotted (1 μL) directly onto the Matrix Assisted Laser Desorption Ionization (MALDI) sample plate, followed by 1 μL of 100 mg/mL norharmane MALDI matrix dissolved in chloroform/methanol/water (3:1.5:0.25, v/v/v). All experiments were performed using a Bruker Autoflex Speed MALDI-TOF/TOF mass spectrometer (Bruker Daltonics Inc., Billerica, MA, USA). Each spectrum was an average of 500 shots and 50% laser power. For MS/MS analysis, precursor ions were chosen and submitted for LIFT TOF/TOF acquisition in the negative ion mode as per Bruker standard MALDI-TOF protocols. ES Tuning Mix (Agilent, Palo Alto, CA, USA) was used as a calibration standard. Norharmane matrix was used throughout these studies.

### 3.4. Outer Membrane NPN Permeability Assay

Outer membrane permeability was determined by using the NPN [31]. Cells were harvested from overnight cultures by centrifugation, washed and resuspended in 20 mM PBS, pH 7.4. The value of OD_600_ was adjusted to 0.5 with the same buffer; the exact OD_600_ was measured and recorded. 80 μL of NPN (1 mM) was quickly mixed with 1.92 mL of the above cell suspension. The fluorescence of the mixture was monitored immediately by a spectrofluorometer (650–600, Hitachi, Tokyo, Japan). The excitation wavelength, emission wavelength and slits used were 350 nm, 420 nm, and 5 nm, respectively. The permeability was indicated by the fluorescence absorption per OD_600_ value of the sample.

### 3.5. Disk Diffusion Assay

The cell culture was incubated at 37 °C until it achieves or exceeds the turbidity of the 0.5 McFarland standard, then a sterile cotton swab was dipped into the adjusted suspension. The Müeller-Hinton agar plate was inoculated by streaking the swab over the entire sterile agar surface. The antimicrobial discs (Remel, Lenexa, KS, USA) were dispensed onto the surface of the inoculated agar plate. The plates were inverted and placed in an incubator at 37 °C. After 16 to 18 h of incubation, each plate was examined. The diameters of the zones of complete inhibition were measured, including the diameter of the disc. Each experiment was repeated three times, and the average values are listed here. The error for the three duplicates was usually less than 2 mm.

### 3.6. THP-1 Cell Stimulations and Measurement of Cytokines

THP-1 cells (200 μL at 2 × 10^5^ cells/mL) were plated in RPMI 1640 medium containing 10% heat-inactivated fetal bovine serum (HyClone, Logan, UT, USA), 2 mM l-glutamine, and 50 nM vitamin D3 (Sigma-Aldrich, St. Louis, MO, USA) in 96-well plates (Corning Costar, Acton, MA, USA) and incubated at 37 °C in humid air with 5% CO_2_ [32]. After 72 h, the medium was replaced with fresh medium containing sonically dispersed LPS ligands or no added stimulus. After 6 h or 24 h of incubation, supernatants were harvested and stored at −80 °C until assayed. 

### 3.7. Stimulation of Mouse MH-S Alveolar Macrophages

Low-passage MH-S alveolar macrophages cells in RPMI 1640 medium containing 10% heat-inactivated fetal calf serum, 10 mM HEPES, 2 mM l-glutamine, 100 U/mL penicillin, and 100 μg/mL streptomycin were cultured at 24- or 48-well plates (Corning Costar, Acton, MA, USA) that had been pretreated with 0.01% poly-l-lysine (Sigma-Aldrich, St. Louis, MO, USA) and incubated at 37 °C in humid air with 5% CO_2_. After 20 h, the medium was replaced with fresh medium containing sonically dispersed LPS ligands or no added stimulus. After 6 h or 24 h of incubation, supernatants were harvested and stored at −80 °C until assayed.

### 3.8. Measurement of Cytokines

Human IL-8, TNF-α and RANTES by THP-1 cells production and MIP-2 by MH-S cells production were measured by enzyme-linked immunosorbent assay (ELISA) 22 h after stimulation using antibody pairs and recombinant standards purchased from R & D Systems (Minneapolis, MN, USA).

## 4. Conclusions

In this study, the effect of acylation pattern of lipid A structure on the permeability of outer membranes of *E. coli* and the innate immune system recognition by human and murine TLR4/MD2 complex have been systematically studied. Six *E. coli* strains which make various numbers of acyl chains of lipid A were constructed, and their outer membrane permeability and innate immune system recognition were analyzed. 

The outer membranes of *E. coli* W3110/pWSK29 showed the least permeability, while mutants of *E. coli* in which lipid A structures were changed by the number of acyl chains showed higher permeability, the less acylated, the more permeable. The permeability analysis suggested that the lipid A acylation pattern serves as good membrane permeability barrier. These results would be useful for designing the membrane structure of industrial microorganism to improve their permeability and increase the yield of products.

The LPS from *E. coli* W3110/pWSK29 showed strong stimulatory in innate immune system recognition by TLR/MD2, while modified penta- tetra- and tri-acylated LPS from *E.* *coli* mutants induced lower level of cytokines, suggesting that all of the modified LPS changed the ability of TLR4/MD2 recognition. The LPS with higher acetylation is more stimulatory than the LPS with lower acetylation. The LPS with lower acetylation is less stimulatory to humans compared with murine TLR4/MD-2. In the same level of acylation, 3-*O*-acyl chain in the lipid A does not serve an important role for TLR4/MD-2 recognition. These results would be useful for designing vaccine adjuvant with less stimulatory response. 

## References

[1] Nikaido H., Vaara M.C. (1985). Molecular basis of bacterial outer membrane permeability. Microbiol. Rev..

[2] Nikaido H. (2003). Molecular basis of bacterial outer membrane permeability revisited. Microbiol. Mol. Biol. Rev..

[3] Raetz C.R., Whitfield C. (2002). Lipopolysaccharide endotoxins. Annu. Rev. Biochem..

[4] Raetz C.R., Reynolds C.M., Trent M.S., Bishop R.E. (2007). Lipid A modification systems in gram-negative bacteria. Annu. Rev. Biochem..

[5] Raetz C.R., Garrett T.A., Reynolds C.M., Shaw W.A., Moore J.D., Smith D.C., Ribeiro A.A., Murphy R.C., Ulevitch R.J., Fearns C. (2006). Kdo_2_-Lipid A of *Escherichia coli*, a defined endotoxin that activates macrophages via TLR-4. J. Lipid Res..

[6] Wang X., Ribeiro A.A., Guan Z., Raetz C.R. (2009). Identification of undecaprenyl phosphate-beta-d-galactosamine in *Francisella novicida* and its function in lipid A modification. Biochemistry.

[7] Li Y., Powell D.A., Shaffer S.A., Rasko D.A., Pelletier M.R., Leszyk J.D., Scott A.J., Masoudi A., Goodlett D.R., Wang X. (2012). LPS remodeling is an evolved survival strategy for bacteria. Proc. Natl. Acad. Sci. USA.

[8] Rebeil R., Ernst R.K., Jarrett C.O., Adams K.N., Miller S.I., Hinnebusch B.J. (2006). Characterization of late acyltransferase genes of *Yersinia pestis* and their role in temperature-dependent lipid A variation. J. Bacteriol..

[9] Hajjar A.M., Ernst R.K., Fortuno E.S., Brasfield A.S., Yam C.S., Newlon L.A., Kollmann T.R., Miller S.I., Wilson C.B. (2012). Humanized TLR4/MD-2 mice reveal LPS recognition differentially impacts susceptibility to *Yersinia pestis* and *Salmonella enterica*. PLoS Pathog..

[10] Guo L., Lim K.B., Gunn J.S., Bainbridge B., Darveau R.P., Hackett M., Miller S.I. (1997). Regulation of lipid A modifications by *Salmonella typhimurium* virulence genes phoP-phoQ. Science.

[11] Groisman E.A. (2001). The pleiotropic two-component regulatory system PhoP-PhoQ. J. Bacteriol..

[12] Gunn J.S., Ryan S.S., van Velkinburgh J.C., Ernst R.K., Miller S.I. (2000). Genetic and functional analysis of a PmrA-PmrB-regulated locus necessary for lipopolysaccharide modification, antimicrobial peptide resistance, and oral virulence of *Salmonella enterica* serovar typhimurium. Infect. Immun..

[13] Trent M.S., Pabich W., Raetz C.R., Miller S.I. (2001). A PhoP/PhoQ-induced lipase (PagL) that catalyzes 3-*O*-deacylation of lipid A precursors in membranes of *Salmonella typhimurium*. J. Biol. Chem..

[14] Kawasaki K., Ernst R.K., Miller S.I. (2004). 3-*O*-Deacylation of lipid A by PagL, a PhoP/PhoQ-regulated deacylase of *Salmonella typhimurium*, modulates signaling through Toll-like receptor 4. J. Biol. Chem..

[15] Casella C.R., Mitchell T.C. (2008). Putting endotoxin to work for us: Monophosphoryl lipid A as a safe and effective vaccine adjuvant. Cell Mol. Life Sci..

[16] Needham B.D., Carroll S.M., Giles D.K., Georgiou G., Whiteley M., Trent M.S. (2013). Modulating the innate immune response by combinatorial engineering of endotoxin. Proc. Natl. Acad. Sci. USA.

[17] Mata-Haro V., Cekic C., Martin M., Chilton P.M., Casella C.R., Mitchell T.C. (2007). The vaccine adjuvant monophosphoryl lipid A as a TRIF-biased agonist of TLR4. Science.

[18] Miller S.I., Ernst R.K., Bader M.W. (2005). LPS, TLR4 and infectious disease diversity. Nat. Rev. Microbiol..

[19] Karow M., Georgopoulos C. (1992). Isolation and characterization of the *Escherichia coli* msbB gene, a multicopy suppressor of null mutations in the high-temperature requirement gene htrB. J. Bacteriol..

[20] Vorachek-Warren M.K., Ramirez S., Cotter R.J., Raetz C.R. (2002). A triple mutant of *Escherichia coli* lacking secondary acyl chains on lipid A. J. Biol. Chem..

[21] Vuorio R., Vaara M. (1995). Comparison of the phenotypes of the lpxA and lpxD mutants of *Escherichia coli.*. FEMS Microbiol. Lett..

[22] Vaara M., Nurminen M. (1999). Outer membrane permeability barrier in *Escherichia coli* mutants that are defective in the late acyltransferases of lipid A biosynthesis. Antimicrob. Agents Chemother..

[23] Park B.S., Song D.H., Kim H.M., Choi B.S., Lee H., Lee J.O. (2009). The structural basis of lipopolysaccharide recognition by the TLR4-MD-2 complex. Nature.

[24] Folch J., Lees M., Sloane-Stanley G.H. (1957). A simple method for the isolation and purification of total lipides from animal tissues. J. Biol. Chem..

[25] Hirschfeld M., Ma Y., Weis J.H., Vogel S.N., Weis J.J. (2000). Cutting edge: Repurification of lipopolysaccharide eliminates signaling through both human and murine toll-like receptor 2. J. Immunol..

[26] Caroff M., Tacken A., Szabo L. (1988). Detergent-accelerated hydrolysis of bacterial endotoxins and determination of the anomeric configuration of the glycosyl phosphate present in the “isolated lipid A” fragment of the *Bordetella pertussis* endotoxin. Carbohydr. Res..

[27] Li Y., Wang X., Ernst R.K. (2011). A rapid one-step method for the characterization of membrane lipid remodeling in *Francisella* using matrix-assisted laser desorption ionization time-of-flight tandem mass spectrometry. Rapid Commun. Mass Spectrom..

[28] Helander I.M., Mattila-Sandholm T. (2000). Fluorometric assessment of gram-negative bacterial permeabilization. J. Appl. Microbiol..

[29] Ernst R.K., Guina T., Miller S.I. (1999). How intracellular bacteria survive: Surface modifications that promote resistance to host innate immune responses. J. Infect. Dis..

[30] Wang R.F., Kushner S.R. (1991). Construction of versatile low-copy-number vectors for cloning, sequencing and gene expression in *Escherichia coli.*. Gene.

[31] Westphal O., Jann K. (1965). Bacterial lipopolysaccharides: Extraction with phenol-water and further applications of the procedure. Methods Carbohydr. Chem..

[32] Six D., Carty S.M., Guan Z., Raetz C.R. (2008). Purification and mutagenesis of LpxL, the lauroyltransferase of *Escherichia coli* lipid A biosynthesis. Biochemistry.

